# Evaluation of short-term outcomes of neoadjuvant chemotherapy followed by radical cystectomy in muscle-invasive bladder cancer: a single Egyptian institution experience

**DOI:** 10.1186/s43046-023-00175-2

**Published:** 2023-05-05

**Authors:** Ibrahim Abdelrahman, Hatem Aboulkassem, Abdallah Elazab, Ahmed Younis Abdallah, Yahia Ismail, Mohammad Taher

**Affiliations:** 1 Surgical Oncology Department, National Cancer Institute, Cairo University, Cairo, Egypt; 2 Medical Oncology Department, National Cancer Institute, Cairo University, Cairo, Egypt

**Keywords:** Muscle-invasive, Bladder cancer, Urothelial carcinoma, Neoadjuvant chemotherapy, NCI-E

## Abstract

**Background and aim:**

Neoadjuvant chemotherapy (NAC) before radical cystectomy (RC) became the standard of care for muscle-invasive bladder cancer (MIBC) in the last few years. We aimed to evaluate the radiological, pathological responses to NAC, and the 30-day surgical outcomes after RC in MIBC.

**Patients and methods:**

A retrospective cohort study involving adult patients with localized urothelial MIBC who received NAC followed by RC at the National Cancer Institute of Egypt (NCI-E) for 2 years (2017 and 2018). Out of 235 MIBC cases, we recognized 72 patients (30%) who fitted the eligibility criteria.

**Results:**

A cohort of 72 patients with a median age of 60.5 years (range 34–87). Hydronephrosis, gross extravesical extension (cT3b), and radiologically negative nodes (cN0) were depicted initially in 45.8, 52.8, and 83.3% of patients, respectively. Gemcitabine and cisplatin (GC) was the rampant NAC employed in 95.8%. Radiological evaluation post NAC using RECIST v1.1 revealed a response rate (RR) of 65.3% in bladder tumor and progressive disease in the former and lymph nodes encountered in 19.4 and 13.9%, respectively. The median time from the end of NAC to surgery was 8.1 weeks (range 4–15). Open RC and ileal conduit were the most common types of surgery and urinary diversion, respectively. Pathological down-staging was encountered in 31.9%, and only 11 cases (15.3%) achieved pathological complete response (pCR). The latter was significantly correlated with the absence of hydronephrosis, low-risk tumors, and associated bilharziasis (*p* = 0.001, 0.029, and 0.039, respectively). By logistic regression, the high-risk category was the only independent factor associated with a poor likelihood of achieving pCR (OR 4.3; 95% CI 1.1–16.7; *p* = 0.038). Thirty-day mortality occurred in 5(7%) patients, and 16(22%) experienced morbidity, with intestinal leakage being the most frequent complication. cT4 was the only significant factor associated with post-RC morbidity and mortality compared to cT2 and cT3b (*p* = 0.01).

**Conclusions:**

Our results are further supporting the radiological and pathological benefits of NAC in MIBC, evidenced by tumor downstaging and pCR. The complication rate after RC is still considerable; hence, more larger studies are necessary to postulate a comprehensive risk assessment tool for patients who would get the maximum benefit from NAC, hoping to accomplish higher complete response rates with ultimately increased adoption of the bladder preservation strategies.

## Highlights

• Muscle-invasive bladder carcinoma needs aggressive treatment beyond surgery alone.

• Neoadjuvant chemotherapy and radical cystectomy trends to be the standard of care.

• Neoadjuvant treatment offered good radiological and pathological responses.

• These responses were shown as downstaging and complete response.

• The hope is to offer complete responders a bladder preservation choice.

## Introduction

Approximately 90% of all primary malignant urinary bladder tumors are transitional cell carcinoma (TCC), which is broadly classified according to the depth of invasion into non-muscle invasive bladder cancer (NMIBC) and MIBC, accounting for about 80–85% and 20–25%, respectively [1]. Squamous cell carcinoma was the predominant form of bladder cancer in Egypt due to bilharzial infestation, however, after successful eradication of the latter, TCC has become the most frequent type over the past 3 decades [2].

Radical cystectomy (RC) with pelvic lymph node dissection (PLND) remains the fundamental treatment approach in localized MIBC. Recently, multimodality bladder-preserving management is considered a possible alternative in a subset of patients [3]. Owing to the presence of non-radiologically detected occult disease at the time of initial diagnosis, employing neoadjuvant chemotherapy (NAC) before RC is now advocated to improve the oncological outcomes [4]. Combination chemotherapy protocols like CMV(cisplatin-methotrexate-vinblastine), M-VAC (methotrexate-vinblastine-cisplatin-doxorubicin) and GC (gemcitabine-cisplatin) have previously demonstrated their effectiveness in advanced and metastatic disease, then have been considered as appropriate regimens in the peri-operative settings [5]. A pivotal meta-analysis of 11 trials including 3005 patients demonstrated a significant survival benefit for the platinum-based NAC with 5 and 9% absolute improvement in 5-year overall and disease-free survival, respectively [6].

The concern of the quality of life (QOL) affection by the use of NAC before RC was addressed in the phase III JCOG0209 randomized trial which compared NAC to upfront RC, no difference in health-related QOL after RC between the two groups was found, that further supports the use of NAC in all eligible patients [7]. The review of the National Cancer Database (NCDB) recommended as fast as possible initiation of NAC in appropriate MIBC patients, but not exceeding 8 weeks after initial diagnosis to avert disease progression [8]. A recent systematic review and meta-analysis by Chung et al. [9] showed a significantly superior rate of pCR and overall survival in patients who received NAC with ddMVAC compared to GC. Consequently, upon a high level of evidence, the NCCN guidelines assigned NAC followed by RC in eligible MIBC patients as a category 1 recommendation [10].

Herein, we present a retrospective 2-year experience of the National Cancer Institute of Egypt (NCI-E) for the short-term outcomes of patients with localized MIBC who received NAC followed by RC.

## Patients and methods

This is a retrospective cohort study involving adult patients aged ≥ 18 years diagnosed with localized muscle-invasive transitional cell carcinoma of the urinary bladder who received NAC followed by RC at NCI-E over 2 years, between January 2017 and December 2018. Exclusion criteria were: initially metastatic disease or localized disease patients who did not receive NAC before RC. After searching the medical records database, 235 MIBC patients presented to NCI-E during the specified time and only 72 (30%) cases were eligible for our study. Patients’ medical charts and pathology slides of the 72 patients were retrieved. All the clinicodemographic data were extracted that included age, sex, residency, medical co-morbidities, smoking history, main presenting complaint, dates of diagnosis, cystoscopic findings and biopsy results, clinical stage at presentation, baseline radiological staging, presence of hydronephrosis, details of NAC, radiologic changes in clinical tumor size and lymph node status after the end of NAC by RECIST Criteria (Version 1.1) [11], surgery date, type and details (urinary diversion method), and post-operative pathological staging. Postoperative 30-day morbidity and mortality according to Clavien-Dindo scale of surgical complications [12]. The TNM staging was according to AJCC staging system 8th edition [13]. Patients were stratified into high and low-risk groups, the former was assigned to patients having baseline hydronephrosis and/or cT3b/T4.

## Statistical methods

Data were analyzed using SPSS version 24. Numerical data were expressed as mean and standard deviation (SD), median, and range as appropriate. Qualitative data were expressed as frequency and percentage. Chi-square (Fisher’s exact) test was used to examine the relation between qualitative variables as appropriate. *p* value ≤ 0.05 was considered significant and all tests were 2-tailed. Multivariate analysis for the factors affecting the pathological complete response (pCR) was done using logistic regression. Odds ratio (OR) with its 95% confidence interval (CI) was used as a risk estimate.

## Results

A total of 72 patients with a median age of 60.5 years (range 34–87), with male predominance (86.1%).The most frequent presenting complaint was hematuria in 77.8% of patients, and less than half (45.8%) had hydronephrosis. Baseline cross-sectional imaging revealed that 52.8% had gross extravesical extension (cT3b), and negative nodes (cN0) in 83.3%; hence, clinical stage III was the most commonly encountered in two-thirds of the patients. As the comment on lymphovascular invasion (LVI) in the initial cystoscopic biopsy pathology reports was not available for most of the cases, patients were considered as high risk if they had baseline hydronephrosis, and/or cT3b/T4 disease, consequently, the majority of patients (79.2%) were assigned to the high-risk group (Table 1).

**Table 1:** Baseline clinicopathological features of 72 localized MIBC patients before receiving NAC

Characteristic/feature	Number	Percent
Age:		
Mean ± SD		
59.2 ± 9		
Median (range)		
60.5 (34–87)		
Gender		
Female	10	13.9
Male	62	86.1
Residency		
Urban	45	62.5
Rural	27	37.5
Smoking history		
No	21	29.2
Yes	51	70.8
Comorbidities		
Yes	24	33.3
No	48	66.7
Comorbidity type (*n* = 24)		
DM	7	29.2
HTN	5	20.8
DM and HTN	4	16.6
HCV	7	29.2
Others	1	4.2
Main presenting complaint		
Hematuria	56	77.8
Necroturia	6	8.3
Dysuria, lower abd pain, others	10	13.9
Hydronephrosis		
Yes	33	45.8
No	39	54.2
Hydronephrosis laterality (*n* = 33)		
Unilateral	19	57.6
Bilateral	14	42.4
Hydronephrosis severity (*n* = 33)		
Mild	13	39.4
Moderate to marked	20	60.6
cT		
Tx	1	1.4
T2	27	37.5
T3b	38	52.8
T4	6	8.3
cN		
N0	60	83.3
N1,2	12	16.7
cTNM stage		
II	23	31.9
IIIA	46	63.9
IVA	2	2.8
Risk category^a^		
Low risk	15	20.8
High risk	57	79.2
Gross cystoscopic findings		
Papillary/fungating	43	59.7
Ulcerative	5	6.9

*cN* clinical nodal stage, *cT* clinical tumor stage, *cTNM* clinical tumor, node, and metastasis stage, *DM* Diabetes mellitus, *HCV* Hepatitis C virus, *HTN* Hypertension

^a^ Based on hydronephrosis and/or cT3/T4

Concerning NAC, the vast majority (95.8%) received Gemcitabine and Cisplatin (GC) as they were fitting the criteria of cisplatin eligibility, Gemcitabine was given as 1 g/m^2^ on days 1 and 8 and Cisplatin as 70 mg/m^2^ on day 1 with the proper hydration every 21 days for 3–4 cycles. The remaining 3 patients (4.2%) were cisplatin-ineligible, one patient received only single-agent Gemcitabine, and the other two received single-agent Carboplatin. The median number of neoadjuvant chemotherapy cycles was 3 (range 1–8), and the majority of the cohort (80.6%) received 3–4 cycles (Table 2).

**Table 2:** Treatment employed, tumors’ response after NAC, and 30-day surgical complications

Category	Number	Percent
NAC type		
Gemceitabine/cisplatin	69	95.8
Gemceitabine	1	1.4
Carboplatin	2	2.8
NAC number of cycles; median (range)		
3(1–8)		
1–2	7	9.7
3–4	58	80.6
5–8	7	9.7
Radiological tumor size response^a^		
CR	3	4.2
PR	44	61.1
SD	11	15.3
PD	14	19.4
Radiological Lymph node response^a^		
PR	12	16.7
SD	50	69.4
PD	10	13.9
Type of radical surgery		
Open radical cystectomy	60	83.3
Laparoscopic radical cystectomy	6	8.3
Anterior pelvic exentration	5	6.9
Robotic radical cystectomy	1	1.4
Type of urinary diversion		
Ileal conduit	53	73.6
Orthotopic neobladder	13	18.1
Cutaneous ureterostomy	4	5.5
Rectal bladder	2	2.8
30-day morbidity/mortality		
No	51	70.8
Morbidity	16	22.2
Mortality	5	7.0
30-day morbidity/mortality causes (*n* = 21)		
Intestinal fistula	7	33.3
Burst abdomen	6	28.6
Renal impairment	3	14.3
Paralytic ileus	3	14.3
Wound infection	2	9.5

*CR* Complete response, *NAC* Neoadjuvant chemotherapy, *PD* Progressive disease, *PR* Partial response, *SD* Stable disease

^a^ By RECIST 1.1

The radiologic clinical response rate by RECIST 1.1 regarding the bladder tumor after completion of NAC was 65.3% (complete and partial responses were found in 4.2 and 61.1%, respectively), whereas progressive disease was depicted in approximately a fifth of the patients (19.4%). Concerning regional lymph nodes, the majority (69.4%) showed a stationary course, meanwhile regressive and progressive diseases were encountered in 16.7 and 13.9%, respectively (Table 2).

The median time between the date of the end of NAC and surgery was 8.1 weeks (range 4–15). Open radical cystectomy was the most commonly employed surgery in 60 patients (83.3%), whereas, laparoscopic radical cystectomy, anterior pelvic exenteration, and robotic radical cystectomy were done in 6, 5, and 1 patients, respectively. The most common method for urinary diversion was the ileal conduit employed in 53 patients (73.6%), whereas orthotopic urinary diversion, cutaneous ureterostomy, and rectal bladder were done in 13, 4, and 2 patients, respectively (Table 2).

Follow-up of patients during the first 30 days postoperatively revealed that mortality occurred in 5 patients (7%) (3 had intestinal fistula with major leak complicated by peritonitis and septic shock, 2 had burst abdomen, after exploration, one of them developed DVT with massive pulmonary embolism, and the other developed severe pneumonia). A further 16 (22%) cases experienced morbidity with intestinal fistula being the most common complication in a third of them (33.3%) (Table 2). Those developed wound infections, have been managed by proper antibiotics with repeated dressings and have recovered smoothly. The patients who developed renal impairment have been managed with proper hydration and fully recovered, apart from one case that needed bilateral percutaneous nephrostomy tubes that were removed after 3 months with a good outcome. The three patients who had paralytic ileus were kept on nothing per oral (NPO) for a longer period, but they recovered without any consequences. According to the Clavien-Dindo grading of complications, G-I and G-III each were encountered in 33.3%, followed by G-V then G-II in 23.8 and 9.6%, respectively (Table 3). Clinical T stage was the only factor that significantly affected the morbidity and mortality in the first 30 days postoperatively, as cT4 was associated with higher morbidity and mortality compared to cT2 and cT3b (*p* = 0.01) in the univariate analysis (Table 4).

**Table 3:** Complications categorised by Clavien-Dindo classification and interventions employed

Complication type	*N* (%) 21(100)	Detailed *N*	Clavien-Dindo grade	Intervention
Intestinal fistula	7 (33.3)	3	V	-Explored then died
		2	IIIb	-Explored then recovered
		2	II	-Antibiotic coverage + TPN (conservation)
Burst abdomen	6 (28.6)	2	V	- Explored, then died (one developed DVT/PE, and one developed severe pneumonia and both died)
		4	IIIb	-Explored then recovered
Renal impairment	3 (14.3)	1	IIIa	-Required nephrostomy tubes, then recovered
		2	I	-Proper hydration (conservation)
Paralytic ileus	3 (14.3)	3	I	- Only conservation (no added treatments to the routinely adopted regimens)
Wound infection	2 (9.5)	2	I	- Only conservation (no added treatments to the routinely adopted regimens)

*DVT* Deep vein thrombosis, *N* Number, *PE* Pulmonary embolism, *TPN* Total parenteral nutrition

**Table 4:** Correlation between clinicopathological features and morbidity and mortality in the first 30 days following radical cystectomy

Morbidity/mortality	No (*n* = 51)		Yes (*n* = 21)		*p* value
	Number	%	Number	%	
Age (years)					
Mean ± SD	59.6 ± 9.3		58.4 ± 8.3		0.795
Gender					
Female	7	70	3	30	0.95
Male	44	71	18	29	
Residency					
Urban	33	73.3	12	26.7	0.55
Rural	18	66.7	9	33.3	
Comorbidity					
No	34	70.8	14	29.2	1
Yes	17	70.8	7	29.2	
Smoking					
Non-smoker	14	66.7	7	33.3	0.68
Smoker	37	72.5	14	27.5	
Hydronephrosis					
Yes	21	76.9	12	23.1	0.22
No	30	63.6	9	26.4	
Hydronephrosis Laterality					
Unilateral	11	57.9	8	42.1	0.33
Bilateral	10	71.4	4	28.6	
Hydronephrosis Severity					
Mild	7	53.8	6	46.2	0.28
Moderate to marked	14	70	6	30	
Clinical T					
T2	20	74.1	7	25.9	**0.01**
T3b	30	78.9	8	21.1	
T4	10	16.7	5	83.3	
Clinical N					
N0	43	71.7	17	28.3	0.73
N1,N2	8	66.7	4	33.3	
Clinical stage					
II	17	37.9	6	26.1	0.82
IIIA	29	72.5	11	27.5	
IIIB	5	62.5	3	37.5	
Risk group					
Low risk	11	73.3	4	16.7	0.81
High risk	40	70.2	17	19.8	
Grade					
II	24	70.6	10	29.4	0.97
III	27	71.1	11	28.9	
Clinical response					
CR/ PR	35	74.5	12	15.5	0.15
SD	5	45.5	6	55.5	
PD	11	78.6	3	11.4	
Downstaging					
Yes	16	69.6	7	30.4	0.87
No	35	71.4	14	28.6	
Associated bilharziasis					
Yes	17	65.4	9	34.6	0.44
No	34	73.9	9	23.1	
Pathological complete response (pCR)					
Yes	8	72.7	3	27.3	0.89
No	43	70.5	18	29.5	
Type of urinary diversion					
Ileal conduit	39	73.6	14	26.4	*
Orthotopic neobladder	9	69.2	4	30.8	
Rectal bladder	1	50	1	50	
Cutaneous ureterostomy	2	50	2	50	

^*^No *p* value because small numbers in subgroups

Pathologic complete response (pT0N0) was achieved in only 11 cases (15.3%), meanwhile, pT3b was depicted in 45.8%. Pathologically positive lymph nodes (pN +) were found in around a fifth of the cases (22%). Down-staging from a higher initial clinical stage to a lower pathological stage was detected in 23 cases (31.9%). Bilharziasis was detected in 36.1%, and the most common associated pathology was squamous differentiation in 44.4% (Table 5).

**Table 5:** Pathological features after NAC followed by radical cystectomy

Feature	Number	Percent
pT		
T0	12	16.7
T1	4	5.6
T2b	6	8.3
T3a	7	9.7
T3b	33	45.8
T4	10	13.9
pN		
Nx	2	2.8
N0	47	65.2
N1,2	23	32.0
LVI		
Yes	15	20.8
No	57	79.2
SM		
Positive	1	1.4
Negative	71	98.6
Associated pathology		
No	39	54.2
Squamous differentiation	32	44.4
Micropapillary pattern	1	1.4
Associated bilharziasis		
Yes	26	36.1
No	46	63.9
Grade		
II	34	47.2
III	38	52.8
pCR		
Yes	11	15.3
No	61	84.7
Down-staging^a^		
Yes	23	31.9
No	49	68.1

*LVI* Lymphovascular invasion, *NAC* Neoadjuvant chemotherapy, *pCR* pathologic complete response, *pN* pathologic nodal stage, *pT* pathologic tumor stage, *SM* Surgical margin

^a^ Comparing the preoperative clinical stage to postoperative pathological stage

**Fig. 1:**
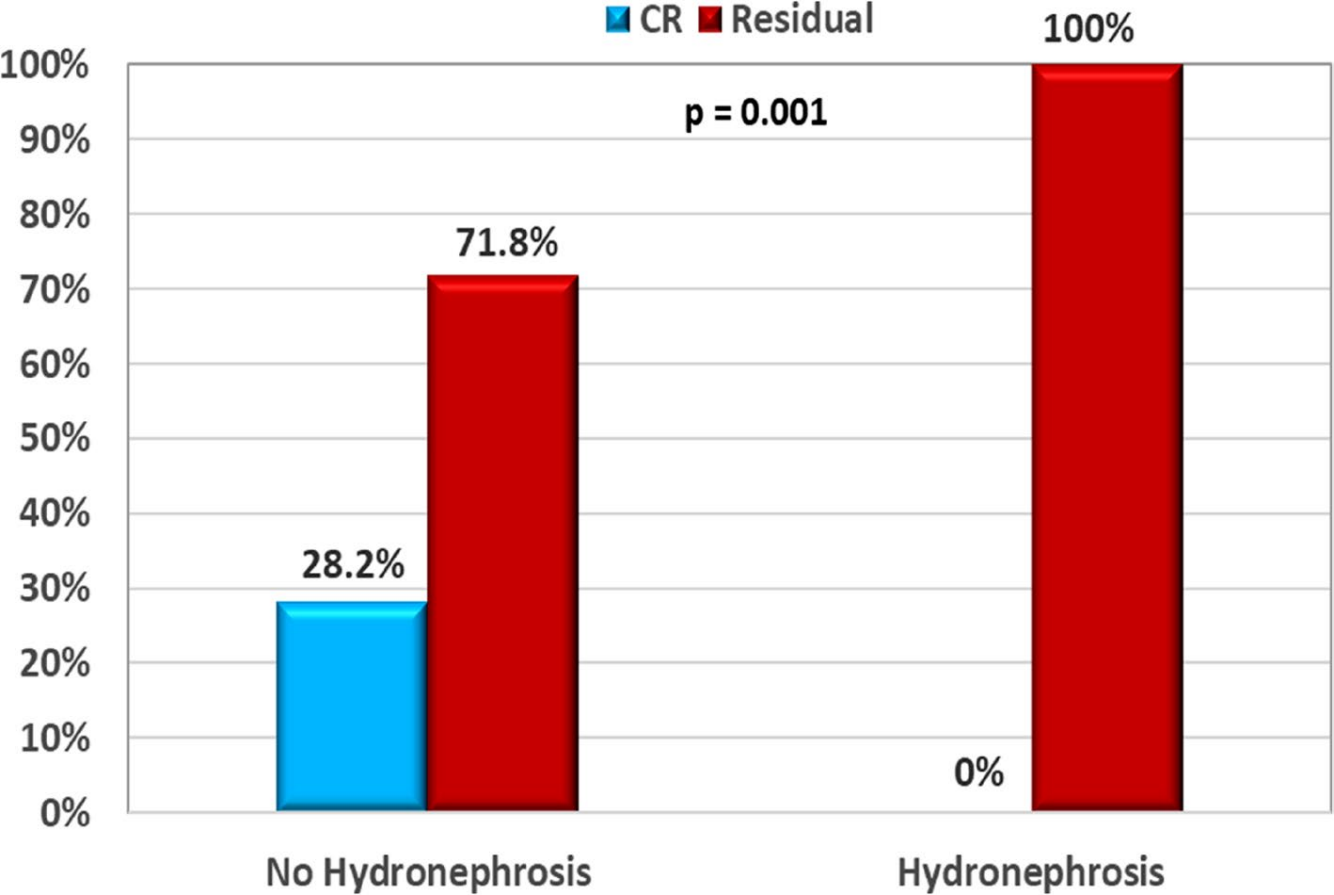
Bar chart showing the relation between presence of hydronephrosis and pCR

**Fig. 2:**
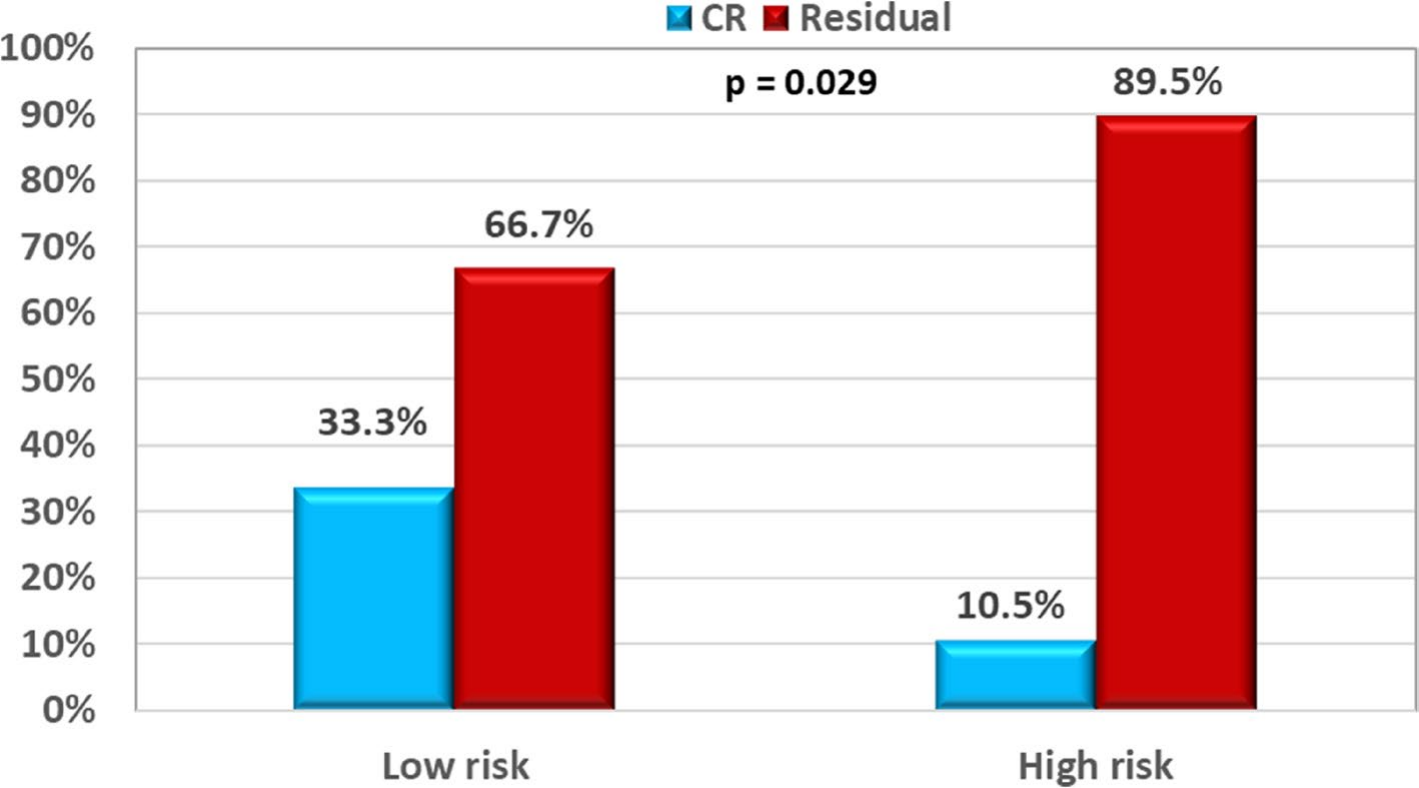
Bar chart showing the relation between baseline risk group and pCR

**Fig. 3:**
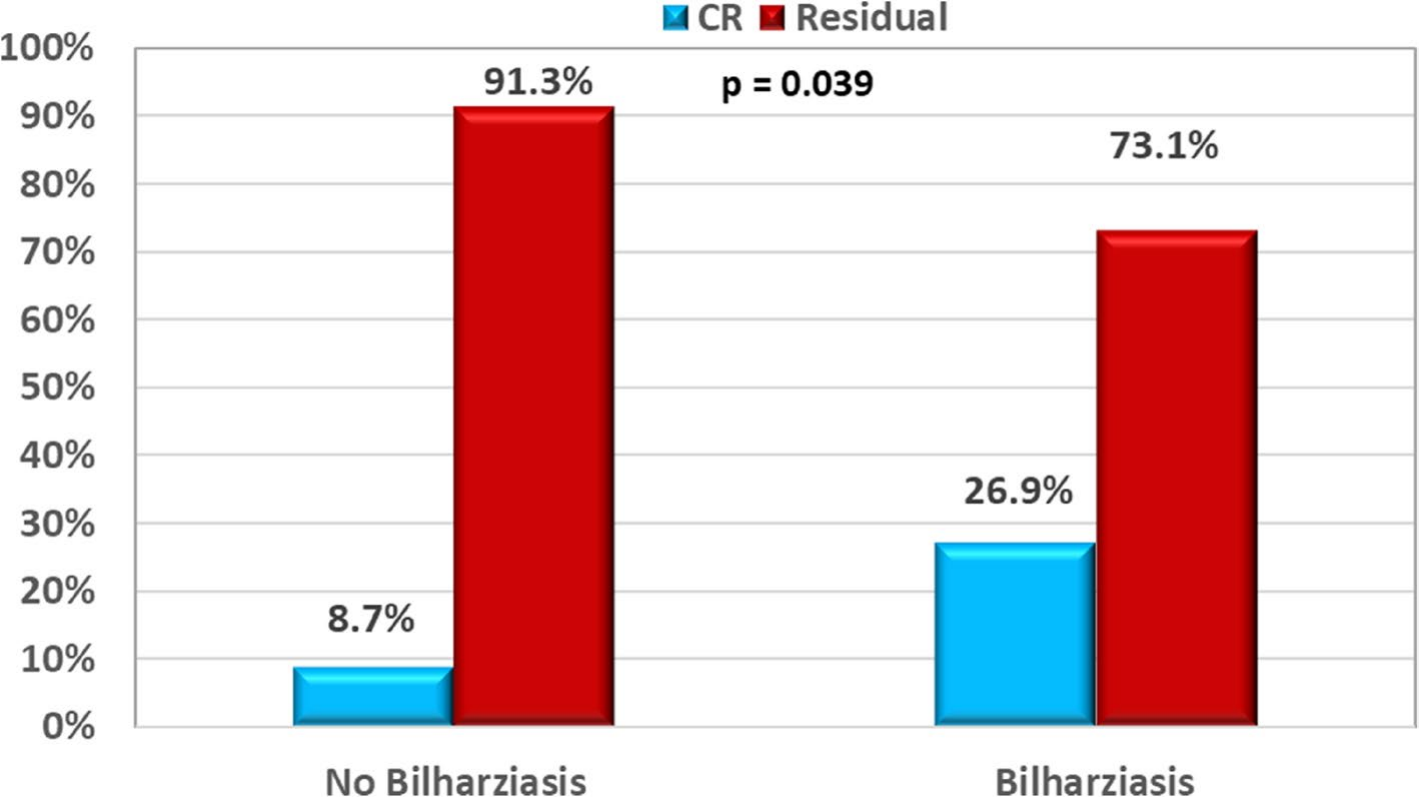
Bar chart showing the relation between associated bilharziasis and pCR

The absence of hydronephrosis, low-risk tumors, and associated bilharziasis were the only factors significantly associated with the achievement of pCR (*p* = 0.001,0.029, and 0.039, respectively) (Figs. 1, 2, and 3). By multivariate logistic regression model, risk category was the only independent factor predicting pCR, as patients with high risk showed 4 times less likelihood of achieving pCR than those with low risk (OR 4.3; 95% CI 1.116.7; *p* = 0.038).

## Discussion

The mean age in the current study was 55.5 years, which is lower than that stated in the reports of Obaid et al. [14] and Anwar et al. [15], (65.8 and 61 years, respectively). These results could be referred to the discrepancy in the sample size and the possible earlier age of presentation of this disease in our country. Concurring with Zarger et al. [16] who reported that the majority of their patients (73.2%) who received NAC had cN0, and 83.3% of our cohort had initially clinically negative nodes, however, in contrast to their results, the major section of our patients (52.7%) presented with cT3b disease compared to cT2 in 69.4% of their cohort.

The pCR after NAC in the current series was associated significantly with the absence of hydronephrosis (*p* = 0.001), exactly matching the results of Poukri et al. [17], these findings could be explained by the conclusions of the work of Resorlu et al. [18], who documented that the presence of hydronephrosis at presentation was associated with advanced pT stage, higher tumor grade, and lymph node metastases. Though we stratified our cohort into high and low-risk subgroups according to only the presence of hydronephrosis and/or cT3b/T4 disease, unlike Moshini et al. [19] who included also LVI and/or associated aggressive histology variants, still patients who were assigned to the high-risk category in our study (79.2%) were higher than what mentioned in their work (55.4%), and that of Lyon et al. (46.9%) [20], this finding might be due to higher initial clinical stage and a higher rate of hydronephrosis in our study than others. Risk class also impacted pCR, as it was significantly higher in those with baseline low-risk features (*p* = 0.029) compared to the high-risk, the latter was the only independent predictive factor for a low likelihood of achieving pCR in multivariate analysis (OR 4.3; 95% CI 1.1–16.7; *p* = 0.038). Von Rundstedt and colleagues [21] stated that NAC was associated with a 1.2 times odds of post-RC risk down-classification due to a greater transformation rate from high to low-risk status in patients treated with NAC compared to patients treated with RC alone.

In respect to NAC type, 96% of our cohort received gemcitabine and cisplatin (GC), adopting the results of the meta-analysis held by Yin et al. [22], which included 1067 patients who received neoadjuvant GC and 667 who received MVAC, no statistically significant difference was observed between the two regimens in respect to the pCR rate (25.7 vs 24.3%, respectively), but GC was less toxic and more convenient to administer, nevertheless, the pCR in our study was only 15.3%, which is lower than that achieved in this meta-analysis, we could refer this finding to the huge difference in the number of patients included, almost two-thirds of our patients had cT3b/4 disease, and 80% were high risk. The majority of our cases (80.6%) received 3–4 cycles, meanwhile, 7 patients (9.7%) received only 1–2 cycles due to poor chemotherapy tolerability, and the remaining 7 cases received a longer course of NAC (5–8 cycles), as their surgery was delayed due to inevitable reasons.

In the current series, the 30-day postoperative complications were experienced in 21(29%) patients, close to what was described by Zakaria et al. [23], who reported that 30.6% of their RC patients suffered at least one complication, and lower than what conveyed by Elmussareh et al. (92%) [24]. The postoperative 30-day mortality in the present cohort was 7% which is higher than what was demonstrated by [23] and [24], (2.8 and 2.2%, respectively), nevertheless, our mortality rate is still within the reported range in the literature (0.8–8%) [25, 26]. Baseline co-morbidities were present in about one-third of the patients who had postoperative morbidity/mortality in our study, however, cT4 was the only factor that was associated significantly with these events compared to cT2 and cT3b (*p* = 0.01) in univariate analysis, whereas, lymphoproliferative disorders and diabetes were significantly associated with severe complications (Clavien-Dindo grade 4–5) in the study of [24].

The authors admit the limitations of this study, being retrospective, including a relatively small sample size as only 72 out of 235 (30%) MIBC patients were candidates for NAC during the specified 2-year period, the comment on LVI in initial biopsy was missed in the majority of the cohort, and the main objective of the study was to evaluate the short-term outcomes of NAC followed by RC, hence, no long-term survival analyses were done.

## Conclusions

Despite the relatively small sample size, our results are further supporting the evidence of the beneficial role of NAC for MIBC before RC in eligible patients, as shown by tumor downstaging and pathologic complete response. Though the latter was experienced in only 15.3%, it was significantly higher in patients without hydronephrosis and with low-risk category. The high-risk class was the only independent predictor factor for a poor likelihood of achieving pCR in multivariate analysis. The complication rate after RC is still considerable; hence, larger-scale studies are mandatory to design a comprehensive risk assessment tool for MIBC patients who would get the maximum benefit from NAC, hoping to accomplish higher complete response rates, with ultimately increased adoption of the bladder preservation strategies.

## Data Availability

The datasets used and/or analysed during the current study are available from the corresponding author on reasonable request.

## Abbreviation

CMV: Cisplatin-Methotrexate-Vinblastine
DVT: Deep vein thrombosis
GC: Gemcitabine-Cisplatin
MIBC: Muscle-invasive bladder cancer
M-VAC: Methotrexate-vinblastine-cisplatin-doxorubicin
NAC: Neoadjuvant chemotherapy
NCDB: National Cancer Database
NCI-E: National Cancer Institute- Egypt
NMIBC: Non-muscle-invasive bladder cancer
NPO: Nothing per oral (os)
pCR: Pathological complete response
PE: Pulmonary embolism
PLND: Pelvic lymph node dissection
QOL: Quality of life
RC: Radical cystectomy
RR: Response rate
TCC: Transitional cell carcinoma
TPN: Total parenteral nutrition
